# Nonlocal Response in Infrared Detector with Semiconducting Carbon Nanotubes and Graphdiyne

**DOI:** 10.1002/advs.201700472

**Published:** 2017-10-25

**Authors:** Zhe Zheng, Hehai Fang, Dan Liu, Zhenjun Tan, Xin Gao, Weida Hu, Hailin Peng, Lianming Tong, Wenping Hu, Jin Zhang

**Affiliations:** ^1^ Center for Nanochemistry Beijing Science and Engineering Center for Nanocarbons Beijing National Laboratory for Molecular Sciences (BNLMS) College of Chemistry and Molecular Engineering Peking University Beijing 100871 P. R. China; ^2^ Collaborative Innovation Center of Chemical Science and Engineering (Tianjin) Department of Chemistry School of Science Tianjin University Tianjin 300072 P. R. China; ^3^ National Laboratory for Infrared Physics Shanghai Institute of Technical Physics Chinese Academy of Sciences 500 Yutian Road Shanghai 200083 P. R. China

**Keywords:** infrared photodetector, s‐SWNTs, uniform response, γ‐GDY

## Abstract

Semiconducting single‐walled carbon nanotubes (s‐SWNTs) are regarded as an important candidate for infrared (IR) optical detection due to their excellent intrinsic properties. However, the strong binding energy of excitons in s‐SWNTs seriously impedes the development of s‐SWNTs IR photodetector. This Communication reports an IR photodetector with highly pure s‐SWNTs and γ‐graphdiyne. The heterojunctions between the two materials can efficiently separate the photogenerated excitons. In comparison to device fabricated only with s‐SWNTs, this IR detector shows a uniform response in the whole channel of the device. The response time is demonstrated to be below 1 ms. The optimal responsivity and detectivity approximately reach 0.4 mA W^−1^ and 5 × 10^6^ cmHz^1/2^ W^−1^, respectively.

Single‐walled carbon nanotubes (SWNTs) have exhibited excellent mechanical, thermal, electrical, and optical properties, and are promising materials in fields including nanoelectronics, photodetector, energy storage, reinforced nanofibers.^1^, ^2^, ^3^, ^4^ In particular, the carrier mobility in semiconducting SWNTs (s‐SWNTs) is up to 10^5^ cm^2^ V^−1^ s^−1^, which guarantees the saturated current density as high as 25 µA per tube.^1^ Owing to the bandgap dependence on their chirality, most of s‐SWNTs show strong and tunable light–matter interaction in the IR region, and have shown great potential in IR detection.^2^, ^3^, ^4^

However, the as‐synthesized SWNTs are usually a mixture of s‐SWNTs and metallic SWNTs (m‐SWNTs), and the metallic nanotubes would accelerate the quenching of excitons. Besides, the relative strong binding energy in s‐SWNTs hinders the separation of photoinduced excitons.^5^, ^6^, ^7^ Those two factors obstruct the development of s‐SWNTs‐based IR photodetection. In the last few decades, the purity of s‐SWNTs can reach 99.9% by several postseparation methods,^8^, ^9^, ^10^, ^11^, ^12^, ^13^ especially by conjugated polymer‐assisted selective dispersion. At the same time, p–n junctions, heterojunctions, and asymmetric electrodes have been employed to promote the dissociation of excitons.^14^, ^15^, ^16^, ^17^, ^18^, ^19^ For example, the performance of s‐SWNTs IR photodetector was improved by using fullerene (C_60_) as an electron acceptor to dissociate excitons.^14^, ^15^, ^16^ In addition, the performance of the photodetector can also be enhanced with asymmetric electrodes, such as scandium (Sc) and palladium (Pd) showing n‐type and p‐type contact with SWNTs, respectively.^17^, ^18^ Though significant progress has been made in the dissociation of excitons, the composite materials will influence the electrical performance of the SWNTs film, for example the on‐state current and carrier mobility usually decrease.^14^, ^15^, ^16^, ^17^, ^18^, ^19^

It is necessary to find a new material that can be combined with s‐SWNTs for enhancing the photoelectric conversion and the meanwhile, maintaining the superior electrical transport property. Among many materials, we found the addition of γ‐graphdiyne (γ‐GDY), a burgeoning material of carbon allotropes with work function of 4.2 eV,^20^, ^21^ can facilitate excitons dissociation. Furthermore, we found that it could maintain the on‐state current and carrier mobility of s‐SWNTs. Here, we report a photodetector fabricated with highly pure s‐SWNTs and few‐layered γ‐GDY. In this device, γ‐GDY plays an important role in the dissociation of excitons in s‐SWNTs film. The energy offset between the lowest unoccupied molecular orbital (LUMO) of γ‐GDY and the conduction band of s‐SWNTs results in the efficient dissociation of excitons. The responsivity and detectivity approximately reached 0.4 mA W^−1^ and 5 × 10^6^ cmHz^1/2^ W^−1^, respectively. More importantly, the device showed uniform photoelectric response covering the entire channel area, instead of the region around electrodes that were shown in previous reports.^7^

**Figure** **1**a shows the structure of s‐SWNTs/γ‐GDY device. A silicon (Si) wafer covered with 300 nm silicon dioxide (SiO_2_) was successively immersed into γ‐graphdiyne and s‐SWNTs solutions. The electrodes were patterned with electron beam lithography and fabricated with 8 nm chromium (Cr) and 60 nm gold (Au) by thermal evaporation. More details are shown in the Experimental Section. From the atomic force microscope (AFM) image in Figure 1b, it is evidenced that uniform s‐SWNT film was formed on γ‐GDY film. The thicknesses of s‐SWNTs film and γ‐GDY film were about 5 and 25 nm, respectively. As shown in Figure 1c, the scanning electron microscope (SEM) image also demonstrated the double layer structure. The block was overlap or fold of γ‐GDY during the deposition. As the Raman spectrum in Figure 1d shows, the peaks at 156 and 178 cm^−1^ are the radial breath modes (RBMs) of s‐SWNTs, which correspond to chiral indexes (15, 7) and (10, 9), respectively. The peak at 2181 cm^−1^ is attributed to the stretching vibration of —C≡C—C≡C— in γ‐GDY.^22^, ^23^, ^24^, ^25^

**Figure 1:**
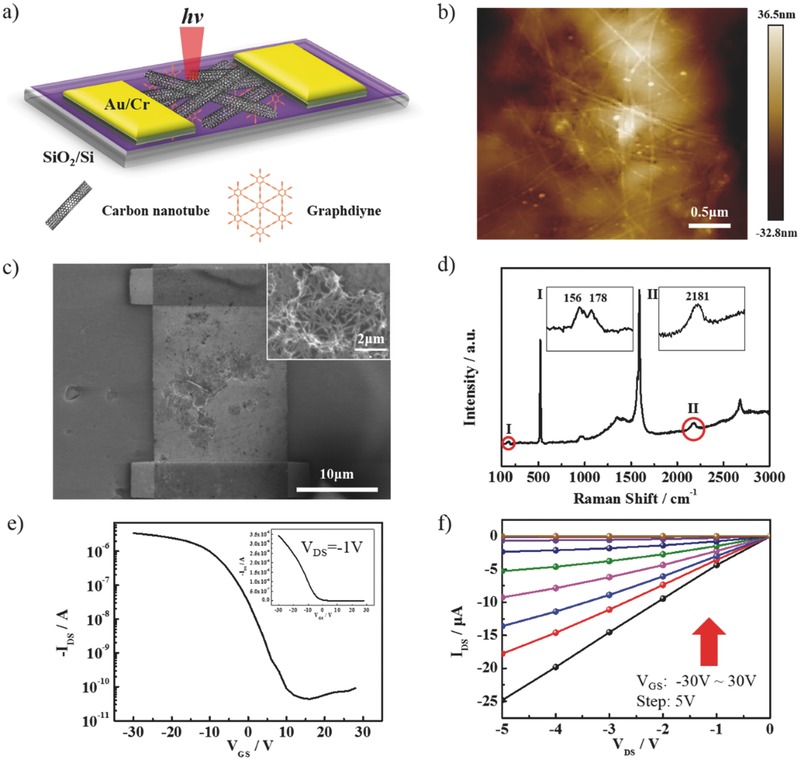
a) Schematic diagram of the device based on s‐SWNTs and graphdiyne. b) AFM image of graphdiyne and s‐SWNTs film. c) SEM image of graphdiyne and s‐SWNTs film, the inset is magnifying image. d) Raman spectra of s‐SWNTs and graphdiyne, the inset I is RBM peak of s‐SWNTs and inset II is the peak of —C≡C—C≡C— in graphdiyne. e,f) Transfer characteristic curve with logarithmic coordinates (inset graph with ordinary coordinates) and output characteristic curve of the device, respectively.

Electrical transport performance was measured on the s‐SWNTs device with a film of γ‐GDY. The transfer curve of the device with drain–source voltage maintained at −1 V and the gate voltage swept from 30 to −30 V was shown in Figure 1e. The on‐off ratio was higher than 3 × 10^5^ and the carrier mobility was about 20.3 cm^2^ V^−1^ s^−1^. The off‐state current was about ≈10^−11^ A. The high on‐off ratio indicated the good semiconducting property, and the low off‐state current indicated a low dark current in IR detection. The mobility was almost the same as the s‐SWNTs device (24.4 cm^2^ V^−1^ s^−1^, shown below). As Figure 1f shows, the current was linear to the drain–source voltage, which implies the Ohmic contact between the materials and the electrodes. These results showed that γ‐GDY can indeed maintain the on‐state current, on‐off ratio, and mobility of the s‐SWNTs film (Figure 3c). In order to show the uniform performance of the s‐SWNTs/γ‐GDY IR photodetector, 50 devices were fabricated, and the uniform response was shown in Figure S1 of the Supporting Information.

The typical photocurrent mapping of the s‐SWNT/γ‐GDY device excited by the 785 nm laser is shown in **Figure** **2**a. It is observed that the photocurrent was uniform in whole channel area with a small bias voltage of 0.5 V. The bandgap of γ‐GDY is 0.46 eV.^19^ Under the irradiation of 785 nm laser, there is no absorption and it was only used to improve the property of s‐SWNTs. γ‐GDY can be seen as p‐type doping of SWNTs, which is evidenced by the shift of the threshold voltage of the transfer curves in Figure 1e and **Figure** **3**c. The energy offset between LUMO of γ‐GDY and the conduction band of SWNTs is greater than the binding energy of excitons, so that the excitons dissociation is facilitated. As a result, the phtotocurrent response in the whole channel was uniform with a bias voltage of 0.5 V. As the inset of Figure 2b shows, the photocurrent with light was 10% larger than the dark current. Furthermore, it is found that the photocurrent continued to increase when gate voltage was applied. The photocurrent became almost three times of the dark current at 30 V gate voltage, which is shown in Figure 2b. On the one hand, the density of carriers could decrease by applying the gate voltage, so that the photocurrent and dark current both decreased; on the other hand, the gate voltage could raise the Fermi level of materials, hence the photocurrent should increase. Thus, the difference between photocurrent and dark current was amplified with increasing gate voltage. During the measurement, the locked current increased with the time as shown in Figure S5 of the Supporting Information. In order to evaluate the performance of the s‐SWNT/γ‐GDY IR photodetector, two typical factors, responsivity and detectivity, were calculated. The responsivity was given by Equation (1) where *I*_s_ is the signal current and *P*_in_ is the incident power density. In our experiment, the incident power was 2 µW. As Figure 2c shows, with the increase of modulation frequency of light, the responsivity (*R*_v_) of the detector was above 0.2 mA W^−1^. It maintained at 0.38 mA W^−1^ when the frequency was under 100 Hz. The detectivity (D*) was near 10^6^ and decreased with the increase of frequency. This can be explained by Equation (2) where *R*_v_ is the responsivity of the device, *A* is the active area of the detector, and *I*_n_ is the noise current. The *I*_n_ increased with the increase of frequency. The *A* of the laser spot was 2 µm. The response time (τ) was shorter than 1 ms as the 3 dB bandwidth provides. The highest frequency here was 1000 Hz, which is limited by our instrument. Usually, 3 dB bandwidth was employed when the response speed cannot catch up with the modulation frequency. The frequency corresponding to half attenuation of the current is the response frequency of the detector. As Figure 2d shows, the half attenuation of the current that should be lower than 0.36 nA appeared after 1000 Hz, which means the τ was at most several hundred microseconds

(1) $$R_{v}\;\;=\;\;\frac{I_{s}}{P_{\mathrm{in}}}$$

(2) $$D*\;\;=\;\;\frac{R_{v}A^{1/2}}{I_{n}}$$

**Figure 2:**
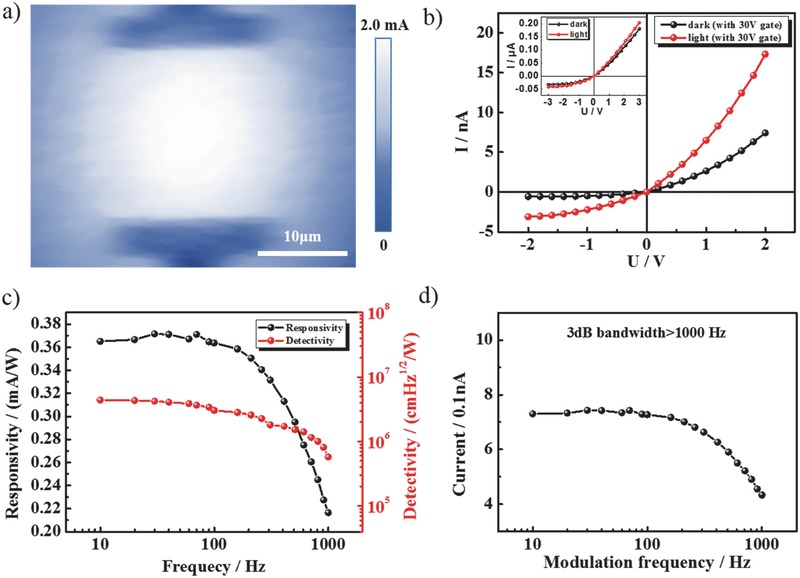
a) IR mapping of the device based on graphdiyne and s‐SWNTs film. b) The *I*–*V* curve of the detector with light on and off at 30 V gate, the inset is *I*–*V* curve without gate voltage. c) Responsivity and detectivity of the device at different frequencies. d) The photocurrent versus switching frequency.

**Figure 3:**
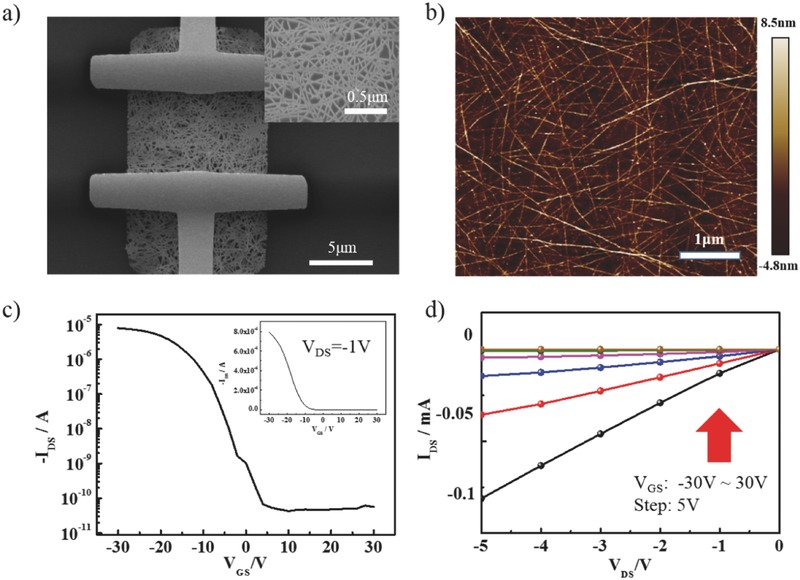
a,b) SEM images of electronic (inset is SEM image of s‐SWNTs film) and the corresponding AFM image of the film, respectively. c,d) Transfer characteristic curve with logarithmic coordinates (inset graph with ordinary coordinates) and output characteristic curve of the electronics, respectively.

To further demonstrate that the photoelectric properties of s‐SWNTs/γ‐GDY device was improved by the introduction of γ‐GDY, a device with only s‐SWNTs was fabricated and tested under the same conditions. As the SEM image in Figure 3a shows, the s‐SWNTs were dip‐coated on a 300 nm SiO_2_/Si substrate. Reactive ion etching (RIE) was employed to remove the redundant SWNTs. The inset shows the density of the s‐SWNTs was similar to the s‐SWNTs/γ‐GDY device. Through the AFM image in Figure 3b, the thickness of the s‐SWNT film was also found to be below 5 nm. The electrical transport property was measured and the transfer curve was shown in Figure 3c. When the gate voltage swept from 30 to −30 V, the on‐off ratio reached 5 × 10^5^ and the carrier mobility was 24.4 cm^2^ V^−1^ s^−1^. The current density of the device approached 1 µA µm^−1^ when the absolute value of drain–source voltage was 1 V. The uniformity was shown in Figure S2 of the Supporting Information. And Figure S3 of the Supporting Information gives statistical data of both devices. The high on‐off ratio was attributed to the high purity of the s‐SWNTs. The output curve shows the s‐SWNTs film and the electrodes also had Ohmic contact, as shown in Figure 3d.

In contrast to s‐SWNTs/γ‐GDY device, the s‐SWNTs device showed obviously lower performance. Because of the high binding energy, the photon excitation can barely contribute to the photoconductivity. Besides, the excitons have short lifetime owing to the quick recombination, so the diffusion length was much shorter. This means that the signal can only probably be collected when the spot irradiated at the contact region between s‐SWNTs and electrodes, which is shown in **Figure** **4**a. The intensity of the signal on two electrodes had no difference and the signal had positive and negative parts at the two electrodes because of the different phases. Owing to the low efficiency, the conductivity could be seen as unchanged between light and dark states. The *I*–*V* curve in Figure 4d indicated the stable conductivity. From the *I*–*V* curve, the resistance of the device can be obtained, which is in the order of magnitudes of 10^4^ Ω. So the photocurrent should be at the order of magnitudes of 10^−11^ A. The current response, which had the same order of magnitudes as the noise current, was too difficult to be measured. Owing to shot noise and 1/*f* noise can be suppressed, the photovoltage was used to evaluate its performance.^17^ As Figure 4b shows, the photovoltage increased with the increase of the incident power and the linear function implies that the device had a stable efficiency. The photovoltage response was related to the S_22_ and S_11_ absorption of s‐SWNT, which correspond to 800–1200 and 1600–2000 nm, respectively, as shown in the inset of Figure 4c. The UV–vis–NIR absorption spectrum also demonstrated the purity of the separated s‐SWNTs. Figure 4c shows the response of the s‐SWNTs film, which shows the obvious photovoltage in the absorption region of the s‐SWNTs. The response was in accordance with optical absorption. When the energy of photon matches the transition energy of s‐SWNTs, it can be absorbed and results in the increase of voltage. To further test the device, the *I*–*V* curve was measured under different conditions. As Figure 4d shows, the current in the dark and global illumination of white light was almost the same. This is because that there was a signal hedge in global illumination of white light. When the light was on, the same intensity but adverse phase response occurred at different electrodes and the signal destructed with each other so that there was no current change with the global illumination of white light. Due to the detect limit of the instrument, the current lower than 0.1 mV cannot be experimentally measured. That explained the nonzero dark current at 0 V. The current increased slightly in the irradiation of 1100 nm laser owing to the low efficiency of the dissociation of photoinduced excitons. The similar phenomenon was also observed in 532 nm laser. The slopes in 532 and 1100 nm laser kept the same, which means the resistance of device did not change under the illumination of different lasers. The invariability of the resistance implies that the photothermoelectric mechanism dominated in the s‐SWNTs device instead of photovoltaic mechanism. It indirectly gives an evidence that the photon excitation had little contribution to the photoconductivity due to the strong dissociation energy of photoinduced excitons.

**Figure 4:**
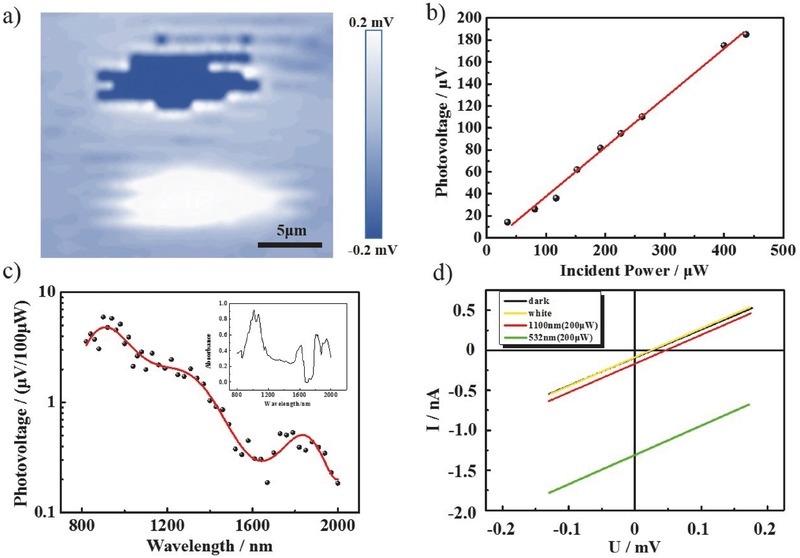
a) IR mapping of electronics based on s‐SWNTs. b) The trend of photovoltage with the change of incident power. c) Response of photovoltage of electronics in near IR field. d) *I*–*V* curve with and without laser.

In general, the low performance of s‐SWNTs device was attributed to the high excitons binding energy of s‐SWNTs. The band diagram depicted in **Figure** **5**a shows that an exciton is formed when s‐SWNTs with work function of 5.0 eV (shown in Figure S4, Supporting Information) absorbs a photon. The electron cannot enter into the conduction band because the interaction between electron (*e*) and hole (*h*) is too strong. The *e*–*h* pair cannot be dissociated and is easily quenched through recombining, which caused the low efficiency. The separated SWNTs were relatively short (about 1–2 µm) and were overlapped in the film. This leads to the low mobility of carriers. In addition to the short lifetime of carriers, the signal can only appear around the electrodes in which the built‐in electric field can help to dissociate the excitons. With the introduction of γ‐GDY, as shown in Figure 5b, the photoinduced electrons can transfer into LUMO of γ‐GDY owing to the small difference between the adjacent energy levels,^16^, ^19^ which promotes the dissociation of excitons and increases the carrier density. When light was on, excitons were generated by absorbing photon and then separated into free carriers with the assistance of γ‐GDY and the small bias voltage so that the signal can be collected by electrodes. Thus, regardless of the position of laser spot, the photocurrent occurred so that response in the whole channel was homogeneous during the photocurrent mapping. Due to the increase of carrier density, the photoelectric response increased.

**Figure 5:**
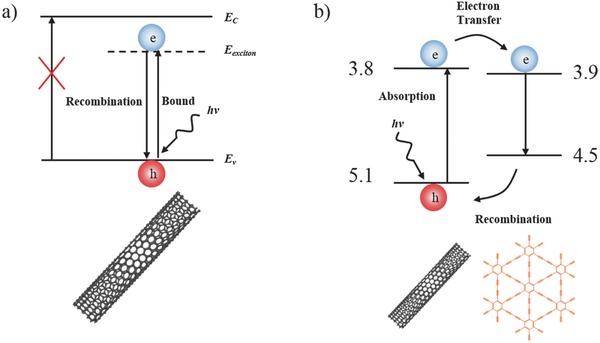
a,b) Schematic of work mechanism of photodetector based on s‐SWNTs only and s‐SWNTs/γ‐GDY, respectively.

In this work, a novel s‐SWNTs/γ‐GDY IR photodetector was fabricated. The device with highly pure s‐SWNTs and γ‐GDY has high on/off ratio exceeding 10^5^ and mobility near 25 cm^2^ V^−1^ s^−1^. The electrical transport property did not decrease compared to s‐SWNTs devices. The introduction of γ‐GDY greatly improved the efficiency of the dissociation of excitons and the s‐SWNTs/γ‐GDY device showed uniform response in the whole channel area. The s‐SWNTs/γ‐GDY photodetector had the responsivity of 0.38 mA W^−1^ and detectivity of 10^6^ cmHz^1/2^ W^−1^. It is believed that the performance of s‐SWNTs/γ‐GDY device can be further improved by using s‐SWNTs of higher quality, such as longer length and uniform orientation, and γ‐GDY of larger scale single crystals.

## Experimental Section

*Preparation of s‐SWNTs and γ‐GDY Film*: The s‐SWNTs were separated with the assistance of a conjugated polymer, poly[9‐(1‐octylonoyl)‐9H‐carbazole‐2,7‐diyl] (PCz), which is described in detail in previous report.^26^ In a typical experiment, 2.50 mg SWNTs, 5.00 mg PCz, and 10.00 mL solvent (8.00 mL toluene and 2.00 mL cyclohexane) were mixed up and sonicated with power of 300 W for 1 h. Then the solution was centrifuged at the speed of 20 000 rpm for 1 h. The purity of sorted s‐SWNTs can reach 99.9%. To remove the PCz wrapped on s‐SWNTs, similar steps were taken as mentioned before. First, the s‐SWNTs solution was filtered with membrane made from polytetrafluoroethylene. Then the s‐SWNTs left on the membrane were washed by toluene for several times followed by ultrasonicated in toluene for 10 min and centrifuged at the speed of 40 000 rpm. γ‐GDY was synthesized by hexaethynylbenzene (HEB) with the catalyst of copper.^22^, ^23^, ^24^, ^25^ First, monomer was obtained from deprotection of precursor hexakis[(trimethylsilyl)ethynyl]benzene by tetrabutylammonium fluoride. The clean copper was put into the mixed solution. Generally, the solution was composed of 100 mL acetone, 5 mL pyridine, and 1 mL tetramethylethylenediamine. Then, 10 mg HEB was added in 50 mL acetone and dropped in it in 3 h. The polyreaction should be carried out at 50 °C for 12 h. And both of the deprotection and reaction should be conducted excluding light. Finally, copper was washed by hot acetone and dimethylformamide and dried in the protection of nitrogen. The as‐synthesized γ‐GDY was dispersed in water with the assistance of sodium deoxycholate.

*Fabrication of s‐SWNTs/γ‐GDY Device*: First, the 300 nm SiO_2_/Si wafer with γ‐GDY and s‐SWNTs was covered by polymethyl methacrylate (PMMA) on spin coater with spin angular speed 2500 rpm for 40 s. Then it was heated at 120 °C for 3 min. And the electron beam lithography was employed to pattern the electrodes area. Finally, the Cr and Au were deposited by vacuum evaporation coating and the PMMA was lifted‐off by acetone.

## Conflict of Interest

The authors declare no conflict of interest.

## Supporting information

Supplementary
