# Supplementary material for: Nonlocal Response in Infrared Detector with Semiconducting Carbon Nanotubes and Graphdiyne
Source: Adv Sci (Weinh). 2017 Oct 25;4(12):1700472. doi: 10.1002/advs.201700472 (PMC5737326; doi:10.1002/advs.201700472)
Supplement: Supplementary file 1 — Supplementary [file ADVS-4-na-s001.pdf]

Copyright WILEY-VCH Verlag GmbH & Co. KGaA, 69469 Weinheim, Germany, 2017.

ADVANCED SCIENCE
Open Access

Supporting Information

for *Adv. Sci.*, DOI: 10.1002/advs.201700472

Nonlocal Response in Infrared Detector with Semiconducting Carbon Nanotubes and Graphdiyne

Zhe Zheng, Hehai Fang, Dan Liu, Zhenjun Tan, Xin Gao, Weida Hu, Hailin Peng, Lianming Tong, Wenping Hu, and Jin Zhang**

Copyright WILEY-VCH Verlag GmbH & Co. KGaA, 69469 Weinheim, Germany, 2016.

Supporting Information

Non-Local Response in Infrared Detector with Semiconducting Carbon Nanotubes and Graphdiyne

Zhe Zheng, Hehai Fang, Dan Liu, Zhenjun Tan, Xin Gao, Weida Hu, Hailin Peng, Lianming Tong, Wenping Hu, Jin Zhang**

1. Materials and Instruments

SWNTs were purchased from Carbon Solution Inc. The 9-(1-octylonoyl)-9H-carbazole-2, 7-diyl was prepared by Suzuki polycondensation in relatively high yield. Toluene and cyclohexane were from Beijing Tong Guang Fine Chemicals company. Hexabromobenzene and tetrabutylamonium fluride (TBAF) was purchased from Alfa Aesar. Copper was from Sinopharm Chemical Reagent Beijing Co., Ltd.

SEM images were obtained by using Hitachi S-4800. AFM measurements were performed using Bruker Dimension Icon. Raman spectra were measured using Horiba LabRAM HR800. The electrical properties were tested using Micromanipulator Keithley MM6200-4200/SCS.

2. Uniformity of electrical transport properties of s-SWNT/γ-GDY devices

To test the stability of s-SWNTs/γ-GDY devices and s-SWNTs devices, we measured 50 devices of each kind. The on-off ratio, mobility and on-state current changed a little among the same kind devices. **Figure S1** and **S2** show representative transfer curves and output curves of former and latter, respectively. **Figure S3** shows the statistical diagram of the devices.

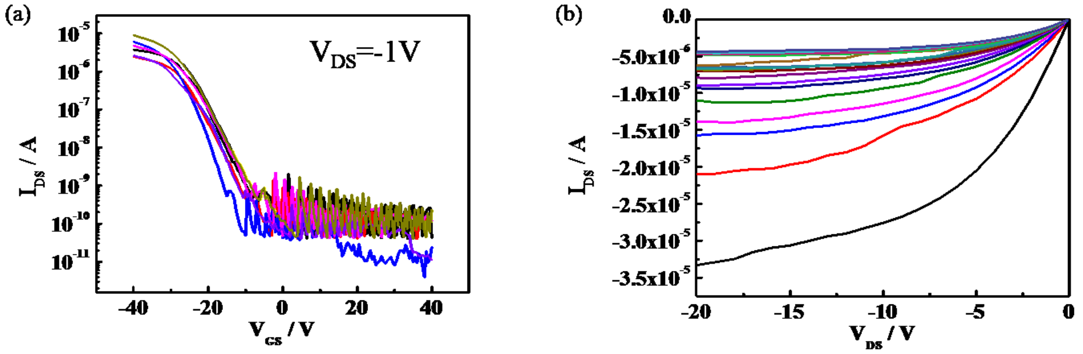

Figure S1 (a) transfer curve and (b) output curve of the s-SWNTs/γ-GDY devices

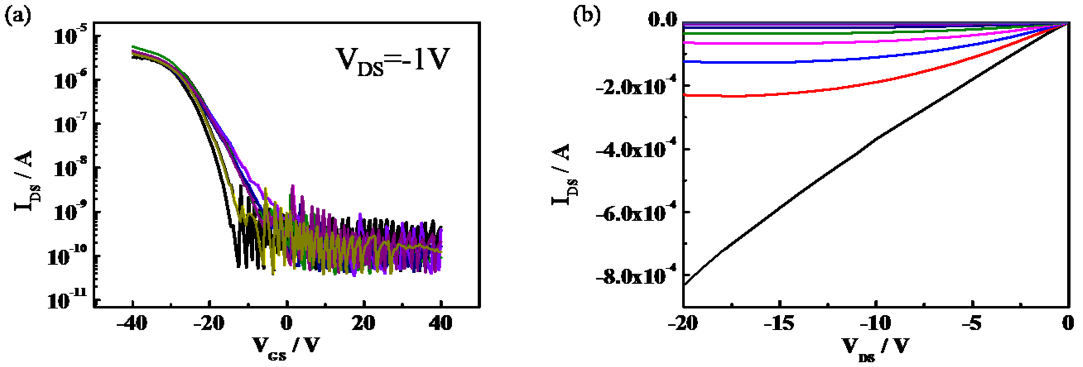

Figure S2 (a) transfer curve and (b) output curve of the s-SWNT devices

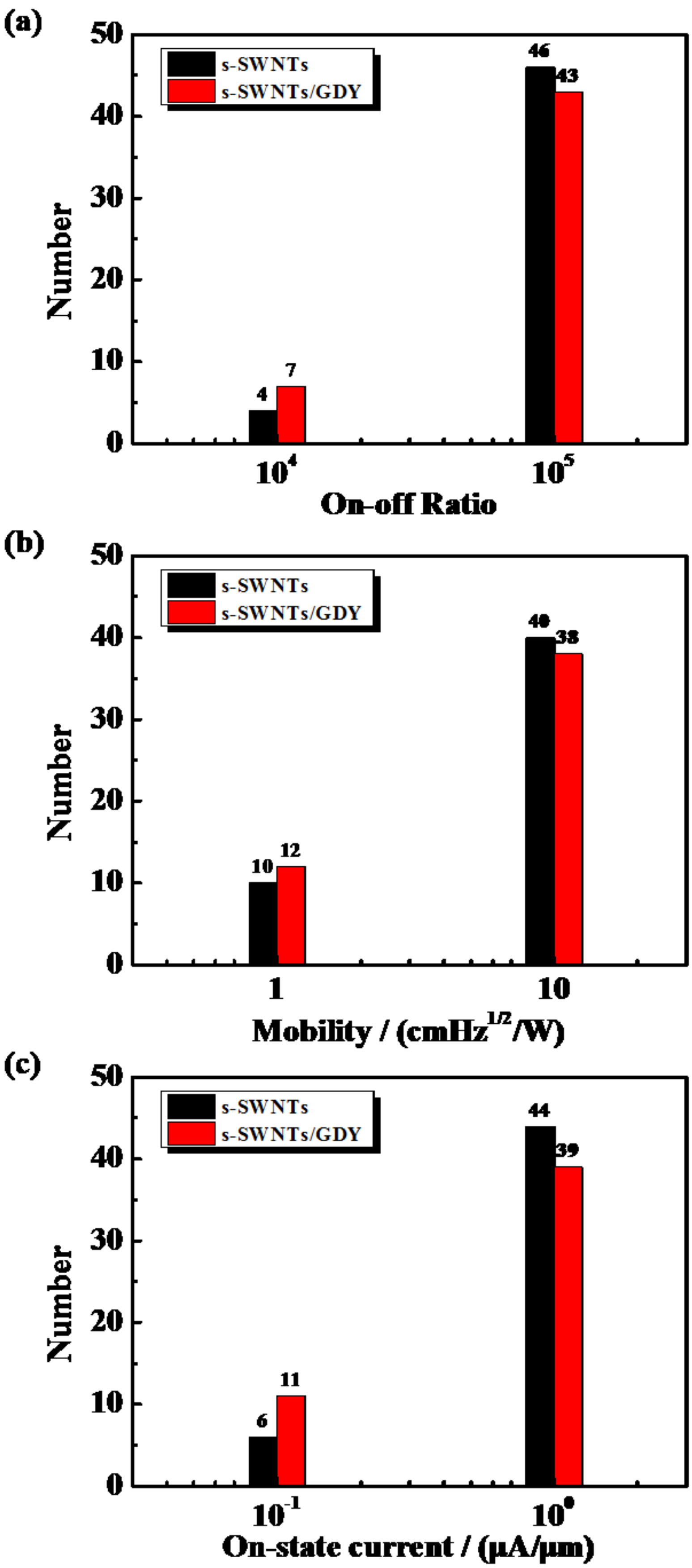
(a)
s-SWNTs
s-SWNTs/GDY
Number
0
10
20
30
40
50
4
7
46
43
10^4
10^5
On-off Ratio
(b)
s-SWNTs
s-SWNTs/GDY
Number
0
10
20
30
40
50
10
12
40
38
1
10
Mobility / (cmHz$^{1/2}$/W)
(c)
s-SWNTs
s-SWNTs/GDY
Number
0
10
20
30
40
50
6
11
44
39
10^{-1}
10^0
On-state current / (μA/μm)

Figure S3 the statistical diagram of s-SWNTs device and s-SWNTs/γ-GDY device in (a) on-off ratio, (b) mobility and (c) on-state current

3. Work function of s-SWNTs

The work function of s-SWNTs was obtained from Kelvin probe force microscopy (KPFM). KPFM can measure the difference between material and stage. We measured the difference between gold electrode as well as s-SWNTs and stage. As **Figure S4** shows, there was a little distinction between each other. The work function of s-SWNT was calculated as 5.0eV.

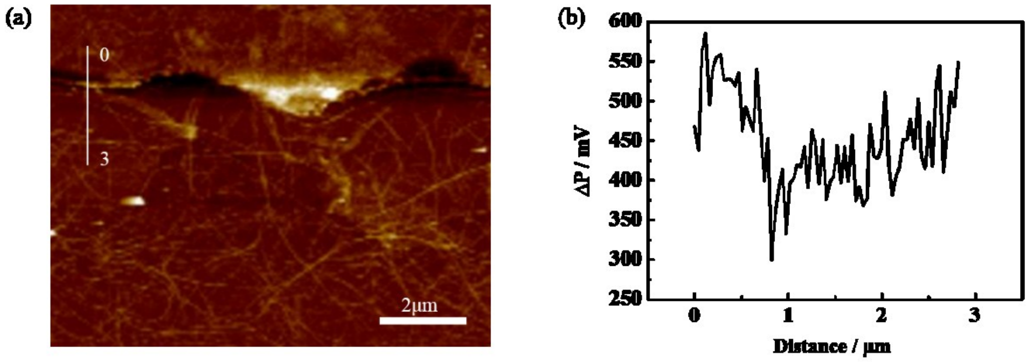

Figure S4 (a) AFM image of gold electrode and s-SWNTs film, location of the white line is corresponding to distance in (b). (b) The difference of the potential between the material and stage.

4. Current shift in IR test

As **Figure S5** shows, during the measurement, the signal locked by lock-in amplifier increased with the time flew, which may be attributed to slow charge transport. Owing to short length of s-SWNTs, there was numerous contact in the film.

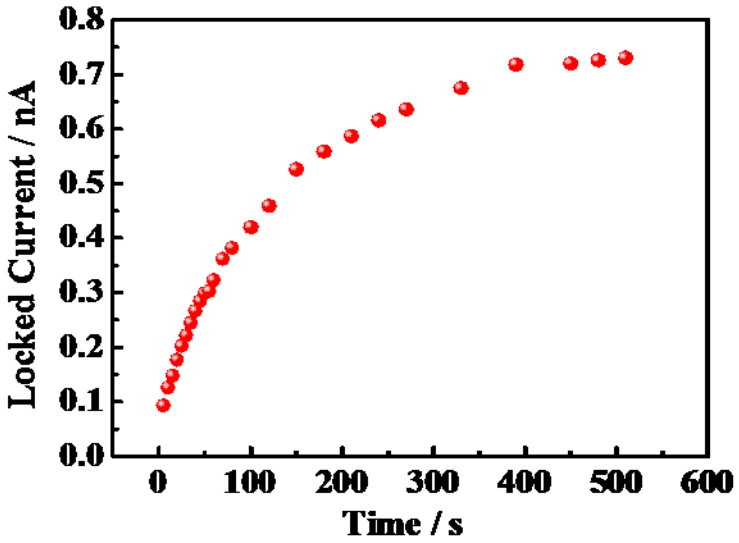

Figure S5 Locked current in the long time measurement

References:

[1] S. Zhang, Y. Hu, J. Wu, D. Liu, L. Kang, Q. Zhao, J. Zhang, *J. Am. Chem. Soc.* **2015**, *137*, 1012.

[2] J. Zhou, X. Gao, R. Liu, Z. Q. Xie, J. Yang, S. Q. Zhang, G. M. Zhang, H. B. Liu, Y. L. Li, J. Zhang, Z. F. Liu, *J. Am. Chem. Soc.* **2015**, *137*, 7596.

[3] X. Gao, J. Zhou, R. Du, Z. Q. Xie, S. B. Deng, R. Liu, Z. F. Liu, J. Zhang, *Adv. Mater.* **2016**, *28*, 168.

[4] R. Liu, X. Gao, J. Zhou, H. Xu, Z. Z. Li, S. Q. Zhang, Z. Q. Xie, J. Zhang, Z. F. Liu, *Adv. Mater.* **2017**, DOI: 10.1002/adma.201604665.

[5] J. Li, Z. Q. Xie, Y. Xiong, Z. Z. Li, Q. X. Huang, S. Q. Zhang, J. Y. Zhou, R. Liu, X. Gao, C. G. Chen, L. M. Tong, J. Zhang, Z. F. Liu, *Adv. Mater.* **2017**, DOI: 10.1002/adma.201700421.
